# Differentiation of Nutritional Risk among Polish Seniors Based on Selected Lifestyle Characteristics

**DOI:** 10.3390/nu14030607

**Published:** 2022-01-30

**Authors:** Robert Gajda, Ewa Raczkowska, Dominika Mazurkiewicz, Edyta Suliga

**Affiliations:** 1 Department of Human Nutrition, Faculty of Biotechnology and Food Science, Wrocław University of Environmental and Life Sciences, 51-630 Wrocław, Poland; robert.gajda@upwr.edu.pl (R.G.); dominika.mazurkiewicz@upwr.edu.pl (D.M.); 2 Institute of Health Sciences, Medical College, Jan Kochanowski University, 25-516 Kielce, Poland; edyta.suliga@ujk.edu.pl

**Keywords:** nutritional risk, elderly, lifestyle

## Abstract

Elderly people living in the community are a population group at high nutritional risk. The aim of this study was to assess the nutritional risk of Polish seniors in relation to the region of residence and selected lifestyle characteristics (alcohol consumption, smoking, sleep, physical activity). The SCREEN-14 (Seniors in the Community: Risk Evaluation for Eating and Nutrition) questionnaire was used to assess nutritional risk. The questionnaire was conducted among 320 Polish adults aged 60 and older. The snowball method was used to select the sample. The study was conducted from May to July 2021 in two regions. Cluster analysis with agglomeration technique was used to identify the association between categories of variables describing nutritional risk level, demographic characteristics, and lifestyle characteristics. Logistic regression analysis was used to assess the association between identified nutritional risk levels and selected lifestyle characteristics. Men over 75 and seniors living in smaller towns have been shown to be at higher nutritional risk. High nutritional risk was significantly more common among those who consumed alcohol, smoked tobacco, and had less than six hours or more than nine hours of sleep per day. In addition, low to moderate physical activity was associated with higher nutritional risk. The identification of factors that increase nutritional risk among seniors can support the planning of ways to prevent nutritional problems in this age group.

## 1. Introduction

Nutritional risk screening among the elderly continues to be of growing interest [1,2,3]. This type of research can contribute to the identification of health risks, effective treatment, and improved health outcomes [3]. It can also form an essential part of service delivery [4].

Differences in the definitions of malnutrition risk and nutritional risk are elusive, despite a recent consensus on the definition of malnutrition [5]. Malnutrition is considered to be a clinical disorder, and malnutrition risk is the term used to describe individuals in a clinical setting who present with indicators suggestive of malnutrition (e.g., very low food intake, weight loss, functional impairment). Most of the screening tools developed to date have focused, at least initially, on identifying the risk of malnutrition in a hospital setting. Nutritional risk, on the other hand, is more general, and involves determinants and risk factors that expose a person to quantitatively and/or qualitatively inadequate food intake which, if not interrupted, may lead to malnutrition [6]. In this context, malnutrition or risk of malnutrition most often affects hospitalized patients and seniors living in nursing homes, whereas nutritional risk is a more common phenomenon among older adults and also affects those living in the community [7].

SCREEN (Seniors in the Community: Risk Evaluation for Eating and Nutrition) is a screening tool used to examine the nutritional risk of community-dwelling older adults [3]. This tool has been adapted [8] and validated [9,10,11] numerous times. Different versions of the SCREEN questionnaire have been shown to be valid for dietary assessment [8] and predictive validity for hospitalization outcomes and mortality [12]. Compared to other nutritional screening tools, SCREEN-14 (previously SCREEN-II) has the highest reliability [13].

Older people living in the community are vulnerable to nutritional risk due to factors that impair food intake [14,15], resulting in poorer health and increased mortality [9,16,17]. The high prevalence of nutritional risk in the elderly is dominated by individual, environmental, and lifestyle characteristics such as being female, living alone, advanced age, low self-rated health, cognitive impairment, functional impairment, depression, dementia, loss of appetite, difficulty in eating food, oral disease, dysphagia, multimorbidity, and low social activity [18,19,20,21,22]. There is a lot of research on malnutrition among the elderly in the available literature, while there is little research on nutritional risk among the elderly. The prevalence of malnutrition in Europe and North America is 1–15% of in-care elderly people, 25–60% of in-care elderly people, and 35–65% of in-hospital elderly people. Nutritional risk assessment is important for the prevention of malnutrition among the elderly, assuming that there is an upward trend in life expectancy. Such an increase in the number of elderly people indicates the need to assess and identify risk factors for malnutrition in the elderly in order to improve their health and quality of life. It is also important to standardize the methods of nutritional risk assessment in order to better compare the obtained research results and to identify more precisely the nutritional risk determinants [19,20].

Studies conducted in different countries of the world using the SCREEN II questionnaire have reported the prevalence of high nutritional risk in between 61.5 and 70.1% of people over 65 years of age [23]. However, few studies have focused on the association of nutritional risk with lifestyle characteristics in older adults [3,20,24]. In addition, the studies carried out thus far have not been conducted in Poland. In this context, the aim of our study was to assess nutritional risk and its differentiation among Polish seniors taking into account selected lifestyle elements.

## 2. Materials and Methods

### 2.1. Study Design and Sample

The survey was conducted between May and July 2021, among people aged 60 and over. The research sample was selected using the snowball method. A total of 900 questionnaires were distributed to 21 clubs, foundations, or other senior citizen organizations in Świętokrzyskie voivodship (city of Kielce, Kielce county and Sandomierz county) and in Dolnośląskie voivodship (city of Wrocław, Olawa county). The study using a direct questionnaire was carried out personally by the authors of the study. The recruitment criterion was residence in the community. Those who agreed to participate in the study were asked to give the questionnaire to people in their place of residence who met the age criterion. In order to reliably assess the nutritional risk depending on selected lifestyle characteristics, subjects following any specific diet recommended by a doctor, dietician. or of their own choice were excluded from the study. A total of 466 surveys were collected, of which 49 were rejected due to missing responses. In addition, 97 questionnaires in which the respondents declared that they were following a specific diet were excluded from the study. The final research sample consisted of 320 people, of which 181 were from Świętokrzyskie and 139 from Dolnośląskie. The study was conducted in accordance with the Declaration of Helsinki [25]. Informed consent was obtained from all participants in the study.

### 2.2. Questionnaire

Table 1 characterizes the questionnaire included in the study.

The SCREEN-14 questionnaire was used to assess nutritional risk [6]. Following the nutritional risk assessment procedure developed for this questionnaire [6], point indices were assigned to each response and then summed. Scores of between zero and four could be assigned for each answer. A score of less than two for a given item indicated the presence of nutritional risk. The maximum score for the SCREEN-14 questionnaire was 64. Based on the cut-off point (50 points), two levels of nutritional risk were distinguished: low nutritional risk (50 or more points) and high nutritional risk (less than 50 points).

Questions on gender, age, place of residence, and region of residence were used to characterize the demographics of the study group.

To assess the association of selected lifestyle characteristics with nutritional risk, questions regarding alcoholic beverage consumption were used; smoking, both in the present and in the past; the number of hours spent sleeping on weekdays and on weekend days; physical activity on weekdays and on weekend days; household composition. In addition, the responses for the question on physical activity allocated to daily activities were characterized as follows: “low physical activity” meant more than 70% of the day spent sitting; “moderate” meant about 50% of the day spent sitting and about 50% moving; and “high” meant more than 70% of the day spent moving.

### 2.3. Statistical Analysis

Qualitative variables are presented as numbers and percentages (%). A chi-square test was used to verify the differences between these variables.

We used a cluster analysis with the agglomeration technique to identify the associations between categories of variables describing nutritional risk level, demographic characteristics, and selected lifestyle traits. In this analysis, a hierarchical classification of variables was applied using Ward’s method, which estimates the distance between sets (clusters) of variables using an analysis of variance [26].

A logistic regression analysis was used to assess the relationship between identified levels of nutritional risk and selected lifestyle characteristics. Odds ratio (OR) values were calculated at the 95% confidence level. Statistical calculations were based on a logistic regression model determined by the formula:

(1) $$P\left(Y=1|x_{1},x_{2},\ldots ,x_{k}\right)=\frac{e^{a_{0}+\sum_{i=1}^{k}a_{i}x_{i}}}{1+e^{a_{0}+\sum_{i=1}^{k}a_{i}x_{i}}}$$

whereby, *a_i_*, *i* = 0,..., *k* are regression coefficients, *x*_1_, *x*_2_,…, *x*_*k*_ are independent variables that can be quantitative or qualitative [26].

The significance level for this value was assessed with the Wald’s test, which checked the statistical significance of regression for the dependent variable [26]. The reference value in the analysis was the value assigned to low nutritional risk. The reference groups (OR = 1.00) were lifestyle characteristics based on declarations of no alcohol consumption, current and past smoking, sleep duration less than seven hours, and low physical activity during the week and at weekends.

Statistical analysis was performed using STATISTICA statistical software (version 13.3 PL; StatSoft Inc., Tulsa, OK, USA; StatSoft, Krakow, Poland) [27].

## 3. Results

### 3.1. Characteristics of the Study Sample

Table 2 shows the demographic characteristics of the study group. Almost three quarters of this group were women and people aged 60–74. Almost two thirds of the respondents lived in a large city, and more than a quarter lived in a rural area. Slightly more than half of the respondents came from Świętokrzyskie province.

Table 3 shows selected lifestyle characteristics for the study group. More than half of the respondents did not consume alcohol and had not smoked tobacco in the past. Nine out of ten respondents declared that they did not currently smoke. More than a half of the respondents stated that they got seven to eight hours of sleep on weeknights and at weekends, while slightly less than a third of the respondents reported six hours of sleep or less per night. About half of the subjects undertook moderate physical activity on weekdays and weekends. Most people reported low physical activity at the weekend and high physical activity on weekdays. Almost two fifths of the respondents lived alone and almost the same with a partner. Almost every tenth respondent lived without a partner but with his family or with a partner and family.

### 3.2. Nutritional Risk

**Figure 1:**
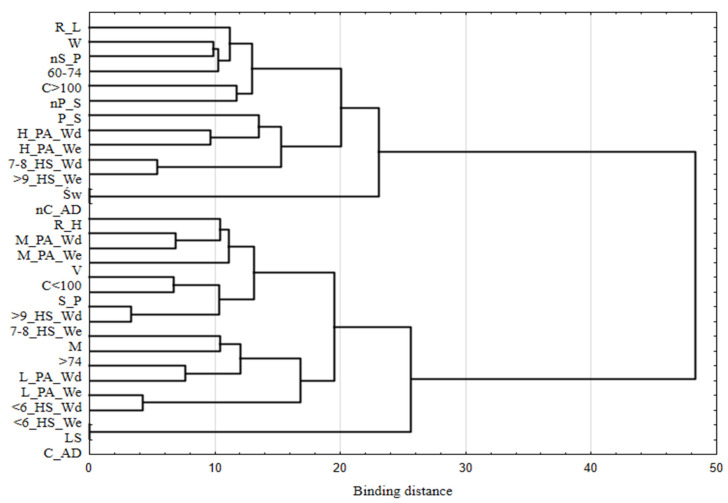
Hierarchical classification of variables describing nutritional risk, based on selected demographic and lifestyle features. R_L—low nutritional risk; R_H—high nutritional risk; W—woman; M—man; 60–74—age in years; >75—age 75 and over; C > 100—city with over 100,000 inhabitants; C < 100—city with up to 100,000 inhabitants; V—village; Św—Świętokrzyskie voivodeship; LS—Lower Silesia voivodeship; C_AD—consumption of alcoholic drinks; nC_AD—no consumption of alcoholic drinks; S_P—smoking in the present; nS_P—no smoking in the present; P_S—past smoking; nP_S—no past smoking; <6_HS_Wd—six hours of sleep or less on weeknights; 7–8_HS_Wd—seven or eight hours of sleep on weeknights; >9_HS_Wd—nine hours of sleep or more on weeknights; <6_HS_We—six hours of sleep or less at weekends; 7–8_HS_We—seven or eight hours of sleep at weekends; >9_HS_We—nine hours of sleep or more at weekends; L_PA_Wd—low physical activity on weekdays; M_PA_Wd—medium physical activity on weekdays; H_PA_Wd—high physical activity on weekdays; L_PA_We—low physical activity at weekends; M_PA_We—medium physical activity at weekends; H_PA_We—high physical activity at weekends.

The structure of the association between the variables describing nutritional risk level and selected demographic and lifestyle characteristics is shown in Figure 1. Using Ward’s hierarchical classification method, we identified two sets of variables (clusters). One set consisted of persons at high nutritional risk, who were characterized by the following features: male sex; age 75 years and over; residence in a village or a small town; residence in Lower Silesia province; alcohol consumption; currently a smoker; six hours of sleep or less on weeknights and at weekends; seven to eight hours of sleep at weekends and nine or more hours of sleep on weeknights; moderate or lower physical activity during the week and at weekends. The second set included persons with low nutritional risk, who were women; aged 60–74; from large cities; from Świętokrzyskie province; did not consume alcohol; did not smoke tobacco either currently or in the past; reported seven to eight hours of sleep on weeknights and nine hours or more at weekends; and reported high levels of physical activity during the week and at weekends.

Almost three quarters of the respondents were characterized as being at high nutritional risk. Alcohol consumption, being a current smoker, getting nine or more hours of sleep on weeknights, and living alone were associated with a significantly higher proportion of those at high nutritional risk. In contrast, low nutritional risk was associated with a significantly higher proportion of those who undertook high levels of physical activity on weekdays and weekends, who reported getting nine or more hours of sleep at weekends, live with a partner, live without a partner but with family, and live with a partner and family (Table 4).

The results of a logistic regression showed that subjects with high nutritional risk were almost twice as likely as subjects with low nutritional risk to consume alcohol (OR = 1.94, *p* = 0.014), more than 2.5 times as likely to be a current tobacco smoker (OR = 2.54, *p* = 0.037) and half as likely to undertake high levels of physical activity on weekdays (OR = 0.51, *p* = 0.000) and at weekends (OR = 0.52, *p* = 0.000). Additionally, unlike people living alone, the nutritional risk was more than two times lower for people living with a partner (OR = 0.44, *p* = 0.000), one third lower for people living with a partner and family (OR = 0.66, *p* = 0.021), and two fifths lower in the case of people living without a partner but with their family (OR = 0.79, *p* = 0.044) (Table 5).

## 4. Discussion

The quality of life of elderly people is determined primarily by physiological changes in the body (e.g., decreased appetite, changes in taste and smell, reduced physical activity), but can also be influenced by psychological (various types of stress, information overload, depressive states), economic (deterioration of material situation), and social aspects (social isolation, lack of recreation and leisure activities with close people, limited mobility). The factors mentioned above may also contribute to increased nutritional risk [28]. Many authors have attempted to assess nutritional risk, mainly among hospitalized patients, and particularly based on anthropometric and blood biochemical indices and dietary analyses [29,30]. Body nutritional status and dietary intake reflect an individual’s health status and facilitate the assessment of nutritional risk. However, few authors have analyzed nutritional risk by taking into account the specific demographic and lifestyle characteristics of people living in the community.

Our study showed that nearly 75% of people over 60 years of age were at high nutritional risk. Similarly, studies carried out in Canada, New Zealand, and the Netherlands have reported high proportions of elderly people at high nutritional risk (61.5–70.1%) [23]. Elements that may have influenced the difference in the proportion of people found in our study to be at high nutritional risk include the final number of participants (320 vs. 13,340); it should be taken into account that our study included only two provinces in Poland, whereas the study by Borkent et al. (2020) [23] examined three countries. In addition, the majority of respondents in our study were 60–74-year olds (73.6%) (Table 2), while in the aforementioned study, 65.8% were 65–74-year olds. In both studies, women made up about three quarters of all respondents. In particular, the factors that contribute to increased nutritional risk and concomitant malnutrition in seniors are psychosocial, environmental, economic, and health-related [31,32,33]. A study conducted by Morais et al. (2013), on the other hand, reported that high nutritional risk was seen in only 25.4% of seniors [34]. However, one should be careful when comparing the results of studies by different authors, as different questionnaires may have been used to assess nutritional risk.

Our results show that gender is a significant factor in increased nutritional risk, with males observed to be at risk significantly more often (Figure 1). The results of studies by other authors also show this relationship. Locher et al. (2005) [35] find that people who live alone or do not have a partner buy lower-quality foods, which may lead to increased nutritional risk. This is particularly noticeable among older men [35], and is primarily due to the fact that among the elderly, there is often a traditional sex-based division of responsibilities in which the woman is responsible for preparing meals. Locher et al. (2005) showed that eating alone is associated with inadequate supply of energy and nutrients, contributing to increased nutritional risk [36]. In addition, among elderly people who experience a change in life situation after the death of their partner, lower food intake is observed due to depression, which is compounded by problems with the independent performance of daily duties [37]. A similar relationship was observed in a study by Tkatch et al. (2020), who found that the duration of meals eaten with family or friends was longer, meaning that older people consumed more and nutritional risk was therefore lower [38]. However, Morais et al. (2013) [34] found that nutritional risk was evenly distributed among men and women, with 50.5 and 49.5% at risk, respectively. The differences from the results of our study are most likely due to the fact Morais et al. did not take into account whether the respondents lived with a partner or alone [34].

In the present study, individuals >75 years of age were shown to be at high nutritional risk. This finding is supported by the results of Wham et al. (2011), who reported that nutritional risk increases with age (among people >85 years of age, it was 85.2%, while in people aged 75–79 years it was 76.6%) [37]. The increase in nutritional risk with age may be due to the fact that people >75 years of age usually live without a partner, and may therefore have reduced motivation to prepare adequate meals in terms of quality and quantity. It has also been shown that eating with other people is 44% higher compared to eating alone [39].

Nutritional risk is related to where the individual lives, and is higher among those living in smaller towns (Figure 1). Crichton et al. (2018) showed that malnutrition that is associated with nutritional risk is more common in rural residents compared to those who live in larger cities [40]. It is also most often associated with poorer health status, lack of support from family [41], and difficulty in accessing health services, usually due to the long distance from home to health care facilities [42].

The present study showed an association between high nutritional risk and lifestyle elements such as alcohol consumption and current tobacco use (Table 4 and Table 5). A study by Damayanthi et al. (2018) [43] conducted among 1015 elderly people in Sri Lanka showed an association of malnutrition and poor nutrition with alcohol consumption and smoking. Alcohol drinkers were four times more likely to have malnutrition (OR = 4.06; *p* = 0.016) [43]. In a study by Mathew et al. (2016) conducted among 113 people over 60 years of age in the Coimbatore area, no relationship was found between malnutrition and the use of stimulants such as tobacco or alcohol [44]. The differences in these findings may be related to the small size of the study group in the study by Mathew et al. Research by Tkatch et al. (2020) [38] showed that 37% of the subjects were characterized as at nutritional risk, whereas in our study the rate was as high as 75%. The authors also observed that those at high nutritional risk were more likely to consume alcohol and smoke cigarettes than those at low nutritional risk. Subjects at nutritional risk consumed alcohol with moderate or high frequency, whereas subjects at low nutritional risk consumed alcohol infrequently or not at all [38].

There is little information in the literature on the relationship between physical activity among the elderly and nutritional risk. Our study showed an association between moderate or low physical activity and high nutritional risk (Table 4 and Table 5). A paper by Al-Zeidaneen et al. (2017) [45] reported that almost half of the elderly people surveyed were at risk of malnutrition, with males being more common. Physical activity was also assessed in the context of malnutrition, and it was observed that only 33% of the respondents reported undertaking physical activity. Malnutrition was more common among physically inactive individuals, whereas moderate or no risk of malnutrition was more common among active individuals [45]. The relationship between physical activity and health and ageing is increasingly being analyzed and described. During the ageing process, the body undergoes numerous changes such as a decrease in lean body mass, including muscle tissue, a loss of muscle strength, and an increase in body fat. Malnutrition can accelerate these processes. A study by Oehlschlaeger et al. (2015) conducted with 210 individuals aged 60+ showed an association between low muscle mass and malnutrition, whereas no correlation was found in the prevalence of malnutrition among seniors and their physical activity [46]. This observation stands in contrast to the results of our study, and may be due to the levels used to categorize physical activity in the study by Oehlschlaeger et al., where seniors were divided into two groups: those who regularly engaged in physical activity and those who engaged only recreationally.

In our study, we observed an association between high nutritional risk and the number of hours of sleep on weeknights and at weekends. Sleep is a biological process that is necessary for proper brain function and body physiology. It has a significant impact on the metabolism, regulation of hunger and satiety sensations, and the endocrine and cardiovascular systems. The quality and quantity of sleep decreases with age. There is little information in the available literature on the relationship between the quality or duration of sleep among older adults and nutritional risk in this group. A study by Jyvacorpi et al. (2020) conducted among men aged 83–99 observed that sleep quality showed a linear trend with nutritional status, as assessed using the Mini Nutritional Assessment Short form (MNA-SF). Seven hours of sleep or less was reported by 32% of the subjects, between seven and nine hours by 51%, and more than nine hours by 17%. Longer sleep was correlated with higher consumption of fish, and overall food intake showed a linear trend with higher sleep quality. Higher vegetable intake was correlated with better sleep quality. In contrast, higher intake of sugars and saturated fatty acids showed a non-significant trend towards poorer sleep quality [47]. The above findings support the claim that a healthy diet can contribute to good sleep among older adults. Research by Kushkestani et al. (2020) [48] conducted among people over 65 years of age found a significant association between age and malnutrition and sleep quality in older subjects. Sleep quality among well-nourished subjects was better than among malnourished subjects. Reduced dietary energy and protein supply or reduced vitamin absorption can lead to impaired body function, which is associated with increased fatigue and reduced sleep quality in older adults [48].

### Strengths and Limitations

As there is no terminological consensus regarding the difference between the terms ‘nutritional risk’ and ‘malnutrition risk’ [5], the original terms from the quoted excerpts from scientific publications were used throughout this text.

The main strength of our study is that it is one of the few nutritional risk assessment studies conducted with the participation of Polish seniors. In our opinion, similar studies should also be conducted in other countries due to differences between national populations. The number of elderly people around the world is increasing, and the results of this study may help in planning the best interventions to reduce nutritional risk, and thus contribute to reducing the burden on health care services. One of the weaknesses of our research is the deliberate choice of two provinces, which was made due to easy access to the research group (as it was the place of residence of the researchers). The results of our survey may therefore not be representative of people over 60 living across the country. Despite the fact that a validated questionnaire was used, our evaluation of the data collected here may be subject to error due to a lack of direct contact with respondents (due to the increased risk of infection from the COVID-19 outbreak, questionnaires were given to facility directors who were willing to participate in the study). In addition, the sample size was small (320 respondents), but this sample size still had sufficient power to detect significant differences between the low and high nutritional risk groups, given the statistical analyses used.

## 5. Conclusions

Depending on their socio-demographic status, older people face different types of nutritional problems. The research has identified non-nutritional factors influencing the occurrence of nutritional risk. Men over the age of 75 and seniors living alone in smaller cities are more likely to be at nutritional risk. High nutritional risk was significantly more common among people who consumed alcohol, smoked tobacco, and slept less than six or more than nine hours a day. Low to moderate physical activity was an additional factor. Identifying and understanding the factors that increase nutritional risk among seniors can help in planning ways to prevent nutritional problems in this age group. The most beneficial approach is to develop strategies to encourage seniors to engage in activities such as preparing and eating meals together, with family or friends, and to undertake physical activity, which can significantly contribute to reducing nutritional risk in this age group. An important aspect involves conveying knowledge about the influence of alcoholic beverages, smoking, and an appropriate amount of sleep on human health.

## Figures and Tables

**Table 1:** Questionnaire characteristics.

Part of the Questionnaire	Survey Questions
The SCREEN-14 questionnaire	Has your weight changed in the past 6 months? Have you been trying to change your weight in the past 6 months? Do you think your weight is….? Do you skip meals? Do you limit or avoid certain foods? How would you describe your appetite? How many pieces or servings of vegetables and fruit do you eat in a day? How often do you eat meat, eggs, fish, cold cuts and legumes? How often do you have milk or milk products such as cheese, yogurt, or kefir? How much fluid do you drink in a day? Examples are water, tea, coffee, herbal drinks, juice, and soft drinks, but NOT alcohol Do you cough, choke or have pain when swallowing food or fluids? Is biting or chewing food difficult for you? Do you use commercial meal replacements or supplements? Examples are shakes, puddings, or energy bars. Do you eat one or more meals a day with someone? Who usually prepares your meals? Which statement best describes meal preparation for you? Do you have any problems getting your groceries? Problems can be poor health or disability, limited income, lack of transportation, weather conditions, or finding someone to shop.
Personal data	Gender Age Place of residence Region of residence What is the composition of your household? Do you consume alcoholic beverages? Do you currently smoke cigarettes, a pipe or other forms of tobacco? Have you smoked cigarettes, a pipe or other tobacco in the past? How many hours per night do you spend sleeping during the week, on average? How many hours per night do you spend sleeping at weekends, on average? How would you rate your physical activity doing everyday activities on weekdays? How would you rate your physical activity doing everyday activities on weekend days?

**Table 2:** Demographic characteristics of the sample.

Variables	*N* = 320	[%]
Gender		
Female	236	73.6
Male	84	26.4
Age		
60–74 years	236	73.6
75 years or older	84	26.4
Place of residence		
Rural area	92	28.8
City < 100,000 residents	25	7.8
City > 100,000 residents	203	63.4
Region of residence		
Świętokrzyskie voivodeship *	181	56.6
Silesia voivodeship	139	43.4

* Świętokrzyskie is the name of a province in southeastern Poland. This name cannot be translated into English.

**Table 3:** Lifestyle characteristics of the sample.

Variables	*N* = 320	[%]
Consumption of alcoholic beverages		
No	181	56.6
Yes	139	43.4
Currently a smoker		
No	296	92.5
Yes	24	7.5
Previously a smoker		
No	183	57.2
Yes	137	42.8
Number of hours of sleep on weeknights		
6 h or less	104	32.5
7 or 8 h	182	56.9
9 or more hours	34	10.6
Number of hours of sleep on weekend nights		
6 h or less	88	27.5
7 or 8 h	189	59.1
9 or more hours	43	13.4
Physical activity on weekdays		
Low	74	23.1
Moderate	173	54.1
High	73	22.8
Physical activity on weekend days		
Low	126	39.4
Moderate	152	47.5
High	42	13.1
Household composition		
I live alone	121	37.8
I live with my partner	124	38.8
I live without a partner but with my family	40	12.5
I live with my partner and my family	35	10.9

**Table 4:** Incidence of nutritional risk in relation to selected lifestyle characteristics.

Variables	Level of Nutritional Risk				*p*
	High		Low		
	*N*	%	*N*	%	
Total	238	74.4	82	25.5	
Consumption of alcoholic beverages					
No	125	52.5	56	68.3	1
Yes	113	47.5	26	31.7	0.047
Currently a smoker					
No	217	91.2	79	96.3	1
Yes	21	8.8	3	3.7	0.041
Previously a smoker					
No	130	54.6	53	64.6	1
Yes	108	45.4	29	35.4	0.963
Number of hours of sleep on weeknights					
6 h or less	81	34	23	28	1
7 or 8 h	131	55	51	62.2	0.998
9 h or more	26	11	8	9.7	<0.001
Number of hours of sleep at weekends					
6 h or less	67	28.2	21	25.6	1
7 or 8 h	141	59.2	48	58.5	0.996
9 h or more	30	12.6	13	15.9	<0.001
Physical activity on weekdays					1
Low	62	26.1	13	15.9	0.986
Moderate	134	56.3	39	47.6	
High	42	17.6	31	36.5	<0.001
Physical activity at weekends					
Low	105	44.1	21	25.6	1
Moderate	109	45.7	43	52.4	0.996
High	24	10.2	18	22	<0.001
Household composition					
I live alone	96	40.3	25	30.5	<0.001
I live with my partner	88	37.3	36	43.9	<0.001
I live without a partner but with my family	29	12.3	11	13.4	<0.001
I live with my partner and my family	25	10.1	10	12.2	<0.001

*p*—significance level (Chi-square test).

**Table 5:** Odds ratio for high versus low nutritional risk with selected lifestyle characteristics.

Variables	Level of Nutritional Risk		
	Low	High	
		OR ^b^	*p*
Consumption of alcoholic beverages			
No	Ref ^a^	1	
Yes		1.94 (1.14–3.12)	0.014
Currently a smoker			
No	Ref	1	
Yes		2.54 (0.70–9.25)	0.037
Previously a smoker			
No	Ref	1	
Yes		1.51 (0.90–2.56)	0.115
Number of hours of sleep on weeknights			
6 h or less	Ref	1	
7 or 8 h		0.73 (0.41–1.29)	0.273
9 h or more		0.96 (0.60–1.53)	0.864
Number of hours of sleep at weekends			
6 h or less	Ref	1	
7 or 8 h		0.92 (0.51–1.66)	0.783
9 h or more		0.85 (0.56–1.28)	0.436
Physical activity on weekdays			
Low	Ref	1	
Moderate		0.67 (0.32–1.36)	0.263
High		0.51 (0.35–0.76)	<0.001
Physical activity at weekends			
Low	Ref	1	
Moderate		0.51 (0.28–0.91)	0.023
High		0.52 (0.35–0.76)	<0.001
Household composition	Ref		
I live alone		1	
I live with my partner		0.44 (0.22–0.88)	<0.001
I live without a partner but with my family		0.79 (0.44–1.30)	0.044
I live with my partner and my family		0.66 (0.30–1.42)	0.021

^a^ reference value; ^b^ odds ratio at 95% confidence level; *p*—significance level for the Wald’s test.

## Data Availability

Data presented in this study are available on request from the corresponding author.
